# Mediastinal Liposarcoma With Anterior and Posterior Mediastinal Involvement: A Thoracic Oncovascular Case Report

**DOI:** 10.7759/cureus.26513

**Published:** 2022-07-02

**Authors:** Papus Keita, Anthony Tran, Mohiuddin Cheema, Nicholas J Peterman, Mario Katigbak

**Affiliations:** 1 Surgery, Carle Foundation Hospital, Urbana, USA; 2 Thoracic Surgery, Baylor Scott and White Health Hospital, Dallas, USA; 3 Surgery, University of Connecticut Health, Farmington, USA; 4 Medicine, Carle Foundation Hospital, Urbana, USA; 5 Thoracic Surgery, Hartford Hospital, Hartford, USA

**Keywords:** innominate vein ligation, vocal cord paralysis, mediastinal tumors, en bloc resection, well differentiated liposarcoma

## Abstract

A 64-year-old patient presented with shortness of breath and chest pressure. The initial examination was unremarkable, and a chest X-ray revealed a large mediastinal mass. Computed tomography (CT) scan demonstrated a lobulated mediastinal mass involving the great vessels and mass effect on the trachea, esophagus, and heart. A CT-guided biopsy showed a monotonous, evenly spaced population of mature, normal-appearing adipocytes consistent with a well-differentiated lipoma-like liposarcoma/atypical lipomatous tumor. The patient underwent a median sternotomy with *en bloc* tumor resection without adjuvant chemoradiation. Three-year follow-up CT imaging shows no evidence of tumor recurrence.

## Introduction

Mediastinal liposarcomas are rare and comprise less than 1% of all mediastinal tumors. The majority are found either in the lower extremities or the retroperitoneum [1]. Diagnosis of these masses is incidentally made during the workup of non-specific symptoms. Presenting symptoms may include dyspnea, dysphagia, cardiomyopathies, and arrhythmias, all depending on the structures involved. On data review, cases of tumor burden involving the anterior and posterior mediastinum concurrently have not been previously published. This case entails a mediastinal liposarcoma with encasement of the great vessels, trachea, and esophagus.

## Case presentation

**Figure 1:**
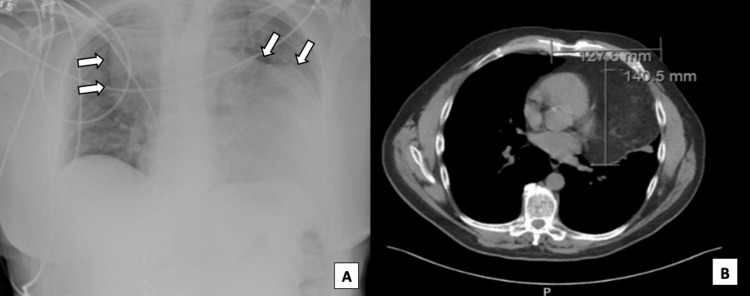
Chest Mass Imaging (A) Anteroposterior X-ray identifying large mass in the chest (white arrows) and (B) axial CT of the chest showing the 127.6 x 140.5 mm mediastinal mass abutting and compressing the left ventricle.

A 64-year-old male complained of dyspnea and intermittent pain and pressure over the chest progressively worsening over months. His history was relevant for gastroesophageal reflux disease (GERD), a hiatal hernia, Factor V Leiden with DVT previously treated with anticoagulation, and a 20-pack year cigarette smoking history. Physical examination revealed a minor reduction in air entry on anterior auscultation and dullness to percussion. He was referred for evaluation of a large mediastinal mass noted on his chest X-ray (Figure 1A).

**Figure 2:**
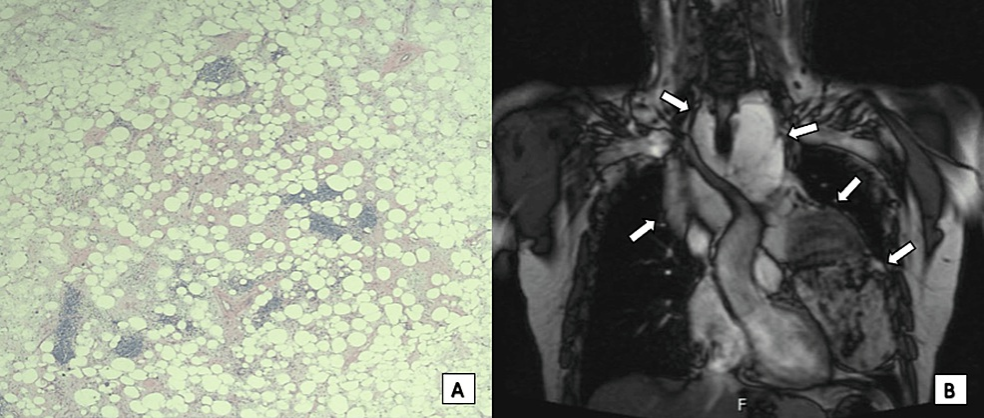
Histology and Magnetic Resonance Imaging (A) Histology showing a monotonous, evenly spaced population of mature, normal-appearing adipocytes consistent with a well-differentiated lipoma-like liposarcoma/atypical lipomatous tumor of the mediastinum and (B) coronal view of magnetic resonance imaging (MRI) of the chest demonstrating the extent of tumor involvement with encasement of the great vessels, innominate vein, superior vena cava, and trachea (white arrows).

A plain CT scan without contrast demonstrated a large lobulated mediastinal mass extending from the thoracic inlet midway to the diaphragm (Figure 1B). The mass encased the trachea causing mild narrowing, as well as posterior displacement of the esophagus. Additionally, the tumor surrounded great vessels, innominate veins, and superior vena cava. The left margin of the heart was also noted to be mildly compressed. A CT-guided biopsy was performed showing a monotonous, evenly spaced population of mature, normal-appearing adipocytes consistent with a well-differentiated lipoma-like liposarcoma/atypical lipomatous tumor (Figure 2A). Chest magnetic resonance imaging (MRI) disclosed a mass measuring 13.2 x 10.4 x 14.4 cm (Figure 2B). Completion oncologic workup with positron emission tomography (PET) -CT did not show evidence of distant disease.

**Figure 3:**
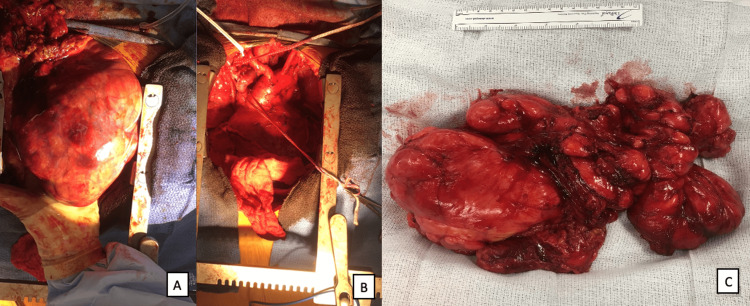
Tumor Visualization (A) Chest cavity with a tumor in vivo and (B) once resected. (C) Specimen of the large anterior and posterior mediastinal tumor after resection.

Following cardiopulmonary evaluations and case discussion during tumor board where potential complications due to surrounding structure operative injury were weighted, the patient underwent a median sternotomy with en bloc resection of the mediastinal sarcoma (Figure 3). Resection required ligation and removal of the involved left innominate vein, phrenic nerve, and recurrent laryngeal nerve. The aortic arch and the arch vessels were explored, and structures were able to be preserved. The postoperative course was complicated by left vocal cord paralysis, right vocal cord paresis, atrial fibrillation, and left upper extremity DVT. Postoperative chest X-ray showed elevation of the left hemidiaphragm consistent with left phrenic nerve resection. The combination of the elevated left diaphragm and vocal cord paralysis raised airway concerns initially, but the patient progressed without any dyspnea or trouble protecting his airway. Formation of left upper extremity DVT was deemed as induced, partly because of Factor V Leiden history and mostly because of innominate vein ligation. The patient was bridged with Heparin-to-Warfarin therapy and discharged on a postoperative day (POD) five.

Following hospitalization, the patient was closely monitored with serial imaging; all were negative for recurrence. He presented 10 weeks postoperatively, complaining of increased shortness of breath. After the appropriate workup, his symptoms were attributed to his previously diagnosed left diaphragmatic hemi-paralysis. He was taken back to the operative room for video-assisted thoracoscopic surgery (VATS) diaphragm plication. There were no complications, and he was discharged on POD one after reporting the resolution of symptoms. To date, he is doing well and is free of any tumor recurrence verified by three-year follow-up imaging.

## Discussion

Liposarcoma of the mediastinum is a rare-occurring entity constituting <1% of all mediastinal tumors as the majority occur in the lower extremities (75%) or the retroperitoneum (20%) [1]. Liposarcomas arise from primitive mesenchymal cells and often originate in adipocytes [2]. As it is such an infrequently encountered diagnosis, there is no standard treatment algorithm [3]. Although there are reports of management using surgery alone versus chemoradiation [4], the efficacy of either treatment or a combination of methods is unknown. When these tumors encase important vascular or airway structures, management becomes even more challenging.

Mediastinal masses can present with various symptoms, including dyspnea, arrhythmias, and dysphagia. These masses usually have an insidious growth course and only become symptomatic once they exert a mass effect on local structures [3]. They are often diagnosed incidentally as part of a workup of cardiopulmonary symptoms [5]. Ultimate management is dependent on specific tumor characteristics, subtype classification, and the extent to which surrounding structures are involved [6]. Since there is limited data available on these tumors, there is no set standard of care, and cases should be evaluated on a multidisciplinary platform. While some studies involving mediastinal masses suggest surgical resection as the best treatment modality [5], others have evaluated pre- and postoperative radiotherapy and chemotherapy; results have failed to show efficacy [7]. Of note, most evidence-based recommendations regarding radiotherapy use have been principally grounded on prospective trials involving extremity soft tissue sarcomas, which generally respond well to function-preserving surgery combined with radiotherapy [8]. 

Liposarcoma of the mediastinum with simultaneous involvement of anterior and posterior compartments is a rare entity that has seldom been described in the literature. The majority of currently described treatment algorithms involve peripherally involved tumors; however, when they are found in the mediastinum, the standard of care is elusive. Reviewing a compilation of the literature helps design the optimal treatment algorithm. Primary surgical resection may be curative for mediastinal liposarcomas, and locally advanced tumors may benefit from neoadjuvant chemoradiation [8]. This case describes a well-differentiated malignant tumor without distant metastasis. Furthermore, the tumor was anatomically favorable for resection despite its involvement of major structures. In circumstances of extensive surrounding tissue involvement where complete resection may not be technically feasible, en bloc debulking has been suggested as the best option [5]. Therefore, the notion that surgical resection alone may be sufficient is supported.

## Conclusions

This report documents a liposarcoma of the mediastinum that achieved a favorable surgical treatment outcome, albeit not without complications involving injury to surrounding structures. The lack of a standard of care for the treatment of this rare tumor necessitates the use of similar case reports and series to supplement clinical decision-making in designing optimal treatment strategies. Despite the involvement of major peri-mediastinal structures, aggressive en bloc resection may be considered in cases without distant metastasis and with anticipation and surgical management of possible resulting complications.
